# The Double Burden: Understanding the Impact of Malnutrition on Childhood Cancer Outcomes in Resource‐Limited Settings

**DOI:** 10.1155/ijpe/8860996

**Published:** 2026-05-11

**Authors:** Michael Asare-Baah, Michael Lauzardo

**Affiliations:** ^1^ Emerging Pathogens Institute, University of Florida, Gainesville, Florida, USA, ufl.edu; ^2^ Division of Infectious Diseases and Global Medicine, College of Medicine, University of Florida, Gainesville, Florida, USA, ufl.edu; ^3^ Keira Grace Foundation, Gainesville, Florida, USA

**Keywords:** cancer outcomes, low- and middle-income countries, malnutrition, nutritional interventions, pediatric cancer, pediatric oncology

## Abstract

Childhood cancer disproportionately affects low‐ and middle‐income countries (LMICs), where over 80% of cases occur, and malnutrition affects approximately 70% of pediatric oncology patients, substantially compromising treatment tolerance, immune function, and survival. This review integrates current evidence on the impact of malnutrition in LMICs, evaluates assessment methodologies and interventions, and identifies knowledge gaps that hinder optimal nutritional care. The A‐B‐C‐D (anthropometric, biochemical, clinical, and dietary assessments) framework provides a systematic assessment, with mid‐upper arm circumference and handgrip dynamometry offering practical evaluation in resource‐limited settings. Prealbumin is the most sensitive early biochemical marker (54%–87% sensitivity vs. 7.4%–45.8% for albumin), and nutritional screening tools like SCAN demonstrate high validity for risk identification (> 80% sensitivity and specificity). Interventions such as ready‐to‐use therapeutic foods improved weight gain (77.8% vs. 64.2%) and reduced severe infections (10.6% vs. 31%) in trials. We emphasize a multidisciplinary approach and research priorities centered on standardized assessments, long‐term effects of malnutrition, and culturally appropriate nutritional programs for LMICs.

## 1. Introduction

Childhood cancer is a major global health problem, with over 80% of incident cases occurring in low‐ and middle‐income countries (LMICs) [1–3]. In these regions, high rates of malnutrition compound the disease burden, negatively impacting care outcomes and survival [4]. While high‐income countries have achieved considerable gains in pediatric cancer survival, LMICs continue to report poorer prognoses. For example, 5‐year survival rates routinely exceed 80% in high‐income countries compared to less than 30% in many LMICs, showing a striking disparity in outcomes. Resource limitations, delayed diagnoses, suboptimal supportive care, and particularly malnutrition are key factors affecting outcomes for pediatric oncology patients in LMICs.

Malnutrition, encompassing both under‐ and overnutrition as well as deficiencies in both macro‐ and micronutrients [4, 5], is a frequent comorbidity in children with cancer, affecting an estimated 70% of patients in LMICs [6, 7]. This nutritional vulnerability stems from the complicated interplay among the disease itself, the catabolic effects of cancer treatment, and the increased physiological demands of growth and development [8]. The pathophysiology of malnutrition in pediatric cancer is complicated. The disease process can directly affect nutrient intake and utilization via mechanisms such as anorexia, nausea, vomiting, and malabsorption [9]. Furthermore, cancer therapies, including chemotherapy and radiation, impose considerable metabolic stress, increasing nutritional requirements while simultaneously compromising the gastrointestinal tract, leading to further nutrient losses and malabsorption [10, 11]. This vulnerability is commonly exacerbated in LMICs, where pre‐existing undernutrition in the general population and poor access to timely diagnosis and appropriate nutritional support contribute to a higher prevalence and severity of malnutrition among pediatric cancer patients [12, 13]. The consequences of malnutrition in pediatric cancers are far‐reaching. Undernutrition compromises immune function, impeding the body′s ability to resist infections and tolerate the rigors of cancer treatment [11, 14]. This could cause increased treatment‐related toxicities, treatment disruptions, and, ultimately, decreased survival rates [15, 16]. Protein–energy malnutrition is the predominant nutritional disorder observed in pediatric oncology, with evidence suggesting that up to 87% of children with cancer exhibit protein deficiency prior to the initiation of therapy [17]. This condition is frequently indicated by anthropometric alterations, such as decreased weight‐for‐age, height‐for‐age, and mid‐upper arm circumference (MUAC), which are optimally evaluated by applying standardized z‐scores that account for age and sex [18]. Micronutritional deficiencies also impair wound healing, delay recovery, and diminish overall treatment efficacy [11]. Specifically, deficiencies in vitamins D, A, C, B complex, and E are commonly observed due to malabsorption, reduced appetite, and modified metabolism induced by cancer and its treatment [11]. Besides physical health, malnutrition negatively affects the quality of life of children with cancer, causing fatigue, psychological distress, and diminished general well‐being [19, 20]. In LMICs, the severity of malnutrition has been directly linked to treatment abandonment [13], further signaling the urgent need for potent nutritional interventions. While the prevalence of undernutrition is widely recognized, overnutrition also entails challenges, increasing the risk of treatment‐related toxicities and long‐term morbidity [16, 21]. Despite the recognized importance of nutritional support in pediatric oncology, considerable barriers exist in LMICs. These include limited access to specialized nutritional products, lack of trained personnel, inadequate educational resources, and financial limitations that often require families to bear the cost of nutritional supplements [22]. Dealing with these challenges is fundamental to improving outcomes.

This review assesses the relationship between malnutrition and pediatric cancer outcomes in LMICs, analyzing links between nutritional status (NS), treatment efficacy, survival, and quality of life. It identifies evidence gaps and research priorities, particularly in the standardization of assessment and interventions, to inform evidence‐based nutritional support for pediatric oncology care in resource‐constrained settings.

## 2. Methods

A systematic literature search was conducted across electronic databases, including Cumulative Index to Nursing and Allied Health Literature (CINAHL), PubMed/MEDLINE, the Cochrane Library, and Google Scholar, following the Preferred Reporting Items for Systematic Reviews and Meta‐Analyses (PRISMA) guidelines. The search involved research studies published between January 2000 and December 2024 to capture research reflecting contemporary diagnostic methods, treatment protocols, and nutritional assessment approaches. Although this timeframe may exclude earlier core studies, it ensures relevance to current clinical practices and incorporates the most recent evidence.

### 2.1. Search Terms and Strategy

The following search terms were combined using Boolean operators: (“protein‐energy malnutrition” OR “protein malnutrition” OR “energy malnutrition” OR “severe acute malnutrition” OR “wasting” OR “stunting” OR “energy‐protein malnutrition”) AND (“childhood cancer” OR “pediatric cancer” OR “pediatric cancer” OR “childhood neoplasm∗”) AND (“LMIC∗” OR “low‐income countr∗” OR “middle‐income countr∗” OR “developing countr∗” OR “resource‐limited setting∗”) AND (“treatment” OR “diagnosis” OR “management” OR “outcome∗”).

### 2.2. Eligibility Criteria

Studies were eligible if they met the following criteria: (1) original research employing quantitative, qualitative, or mixed method designs, (2) focused on malnutrition in pediatric cancer patients aged 0–18 years, (3) conducted in LMICs as recognized by the World Bank classification, (4) published in peer‐reviewed journals between January 2000 and December 2024, and (5) published in English or with English translations available. Studies conducted in high‐income or upper middle‐income countries were excluded. Conference abstracts, case reports, and opinion pieces were also excluded.

### 2.3. Study Selection Process

**Figure 1:**
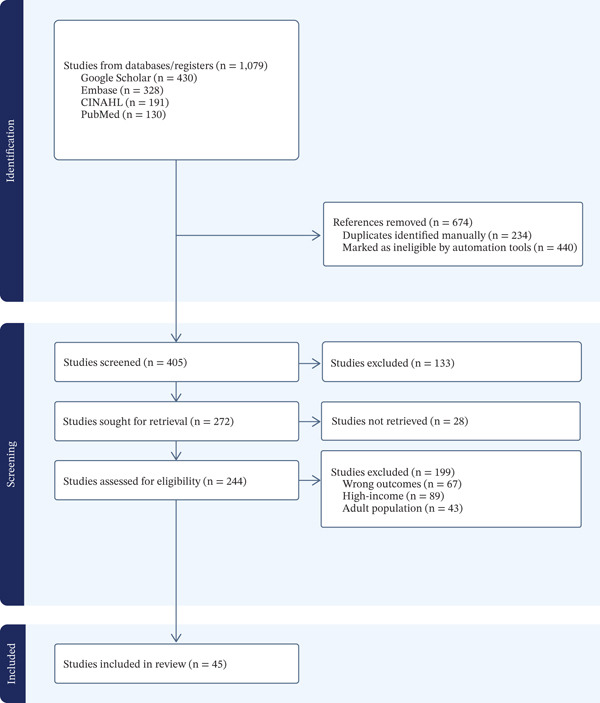
PRISMA flow diagram for study selection. Studies were excluded for the following reasons: conducted in high‐income countries (*n* = 67), nonoriginal research/review articles (*n* = 38), not focused on malnutrition outcomes (*n* = 18), and inadequate sample characterization (*n* = 10). Studies not retrieved (*n* = 28) were mainly due to paywall restrictions without institutional access (*n* = 19) and to full‐text availability despite attempts to contact the authors (*n* = 9).

Two independent reviewers (MAB and ML) screened titles and abstracts for relevance using a standardized screening form developed in the Covidence online software. Full text of potentially eligible studies was retrieved and independently assessed against the inclusion criteria. Studies not retrieved (*n* = 28) were primarily inaccessible due to institutional access limitations (*n* = 19) or were unavailable despite direct author contact (*n* = 9). Inconsistencies were resolved by consensus. Review studies were documented using a PRISMA flow diagram (Figure 1).

## 3. Results

### 3.1. Overview of Reviewed Literature

The body of evidence reviewed is predominantly focused on acute lymphoblastic leukemia (ALL), with studies concentrated in a limited number of regions and cancer types, emphasizing considerable gaps in the literature from underrepresented settings and diagnoses. The 45 included studies comprised observational cohort studies (*n* = 23), cross‐sectional analyses (*n* = 12), randomized controlled trials (*n* = 6), and qualitative investigations (*n* = 4), conducted across 18 LMICs (Table 1). Geographic distribution included sub‐Saharan Africa (*n* = 19), South Asia (*n* = 14), Latin America (*n* = 9), and Southeast Asia (*n* = 3). Predominant cancer types investigated were ALL (62%), solid tumors (24%), and mixed diagnoses (14%).

**Table 1:** Summary of reviewed literature (*n* = 45 studies).

Characteristic	*n*(%)
Study design	
Observational cohort	23 (51.1)
Cross‐sectional	12 (26.7)
Randomized controlled trial	6 (13.3)
Qualitative	4 (8.9)

Geographic region	
Sub‐Saharan Africa	19 (42.2)
South Asia	14 (31.1)
Latin America	9 (20.0)
Southeast Asia	3 (6.7)

Primary cancer type	
Acute lymphoblastic leukemia	28 (62.2)
Solid tumors	11 (24.4)
Mixed diagnoses	6 (13.3)

Thematic focus	
Prevalence/correlates of malnutrition	26 (57.8)
Assessment methodologies/tools	13 (28.9)
Intervention efficacy	6 (13.3)

Sample size	
< 50 participants	18 (40.0)
50–150 participants	19 (42.2)
> 150 participants	8 (17.8)

The literature synthesis identified three primary thematic domains: (1) prevalence and correlates of malnutrition at diagnosis (*n* = 26 studies), (2) nutritional assessment methodologies and tool validation (*n* = 13 studies), and (3) intervention impact and implementation (*n* = 6 studies). Notable gaps include the lack of uniform assessment protocols, limited data on long‐term survivor outcomes, and insufficient scalable intervention models for resource‐limited settings. Few studies address the mechanical pathways linking malnutrition to treatment tolerance and survival, as most focus primarily on prevalence documentation rather than intervention evaluation.

### 3.2. Nutritional Screening in Pediatric Oncology

As part of a comprehensive nutritional assessment, systematic screening tools facilitate early identification of at‐risk patients who require detailed evaluation. The Nutrition Screening Tool for Childhood Cancer (SCAN) demonstrates validity in identifying malnutrition risk across diverse cancer diagnoses, with sensitivity and specificity exceeding 80% [23]. Comparative assessments indicate that SCAN outperforms generic pediatric screening tools in oncology‐specific risk detection, particularly in identifying patients who require intensive nutritional intervention [24–26]. Implementation of standardized screening guidelines in LMIC settings enables resource‐efficient triage, directing intensive assessment toward high‐risk patients while continuing systematic monitoring across all cases. Despite demonstrated utility, extensive adoption remains limited in resource‐limited environments due to training requirements and competing clinical priorities [27].

### 3.3. Causes and Development of Malnutrition in Children With Cancer

Reviewed studies revealed that the pathophysiology of malnutrition in pediatric cancer is multifactorial, extending beyond cytokine‐mediated cachexia to involve the gut microbiome, chronic inflammation, and treatment‐associated metabolic disturbances [28]. Early disruption of the gut microbiome and persistent dysbiosis, in combination with elevated proinflammatory cytokines such as TNF‐*α*, IL‐1*β*, IL‐6, and IFN‐*γ*, establishes a chronic inflammatory state that impairs nutrient absorption and utilization [29]. These cytokines not only accelerate the mobilization and oxidation of energy substrates, leading to increased lipolysis and loss of whole‐body proteins, but also directly impact central nervous system pathways that regulate appetite and energy expenditure [30, 31]. In addition, treatment‐induced metabolic alterations further disrupt protein synthesis, energy metabolism, and chemotherapy pharmacokinetics, thereby worsening the nutritional crisis [32].

### 3.4. Assessing NS in Children With Cancer

NS assessment is an essential part of comprehensive care for pediatric cancer patients. The choice of assessment method can influence the prevalence of malnutrition identified [33, 34]. The A‐B‐C‐D method has been recognized as a standardized and effective tool for NS screening in high‐risk populations [8]. This method encompasses four key components: anthropometric measurements (A), biochemical examinations (B), clinical evaluations (C), and dietary intake assessments (D), presenting a holistic approach to nutritional care [8].

Anthropometric assessment (A): Anthropometric measurements form the foundation of NS evaluations. This component measures physical dimensions, including weight, height, body mass index (BMI), and MUAC [34]. Anthropometric monitoring shows clear patterns during cancer treatment. In the early phases of treatment, BMI z‐scores typically increase, with meta‐analysis data showing a mean increase of 0.8 in pediatric patients with ALL [35]. However, MUAC is more sensitive than BMI for detecting malnutrition, especially in patients with solid tumors, where tumor mass may obscure weight‐based assessments. Studies indicate that MUAC identifies 12.3%–30.3% of children as undernourished, compared to BMI′s detection rate of only 5.2%–14.7% in the same populations [34, 36].

Muscle strength assessment, particularly handgrip dynamometry, provides complementary functional data regarding NS [37, 38]. Recent research highlights handgrip strength as an early clinical “vital sign” for nutritional risk, with the potential to serve as an advanced warning indicator of declining functional status. Studies demonstrate significant associations between reduced handgrip strength and malnutrition in pediatric oncology patients, with strength measurements linked to both anthropometric indices and clinical outcomes [37]. Importantly, handgrip strength not only reflects current nutritional deficits but also predicts treatment tolerance and the risk for functional mobility impairment in children and adolescents with cancer [38]. This prognostic capacity positions routine handgrip measurement as a useful inclusion to assessment protocols, encouraging earlier intervention. Unlike cutting‐edge imaging modalities such as dual‐energy x‐ray absorptiometry (DEXA), handgrip dynamometry represents a practical, cost‐effective option readily implementable in LMICs for monitoring functional NS. These measurements are important for identifying variations from expected growth patterns, which may indicate malnutrition or treatment‐related complications. In pediatric oncology, regular anthropometric and functional assessments are essential for monitoring growth trajectories and changes in body composition during treatment.

Biochemical assessment (B): Biochemical tests provide valuable insights into the metabolic state of pediatric cancer patients. These assessments involve laboratory tests to evaluate protein status, organ function, bone health, anemia, inflammation, and specific mineral and vitamin deficiencies. Among the biochemical markers evaluated across the reviewed studies, prealbumin displayed superior sensitivity as an early malnutrition indicator compared with albumin, with deficiency detected in 54%–87% of patients at diagnosis, versus 7.4%–45.8% for albumin [17, 39]. Emerging techniques, such as the use of salivary biomarkers, offer a less invasive means of assessing NS [40–42], though validation in pediatric oncology populations remains limited. In cancer treatment, biochemical assessments are particularly important for monitoring the effects of chemotherapy and radiation on nutrient absorption and metabolism.

Clinical evaluation (C): Clinical evaluation is a key element of the A‐B‐C‐D method, allowing the identification of overt signs of malnutrition and treatment‐related side effects. This extensive assessment includes the evaluation of subcutaneous fat (loss or excess), muscle wasting, and changes in the skin and hair [8]. Furthermore, recent weight changes, edema, dryness of mucous membranes, and signs of vitamin and mineral deficiencies are carefully documented [8]. Given the possible impact of cancer treatment on NS, it is essential to assess treatment‐related side effects that may compromise oral intake. These side effects can include nausea, vomiting, anorexia, diarrhea, constipation, flatulence, belching, indigestion, mucositis, dysphagia, taste alterations, xerostomia, and impaired chewing and swallowing abilities [8]. Clinical evaluation provides a complete view of a patient′s health through integrating findings from other assessments to guide nutritional interventions.

Dietary assessment (D): Dietary assessment is necessary to evaluate nutrient intake and identify areas for intervention. Techniques such as 24‐h recall, food frequency questionnaires (FFQs), and food diaries are used to gather detailed information about patients′ dietary habits. This component is particularly important in pediatric cancer patients, who may have increased nutritional requirements due to their disease and treatment. Understanding dietary patterns allows health practitioners to recommend specific dietary variations to support treatment and recovery. Moreover, dietary assessments can inform counseling on grocery shopping, food hygiene, storage, preparation, and serving practices, aligned with WHO‐approved food safety guidelines that take into account resources in LMIC settings [43, 44].

### 3.5. Interventions and Management Strategies

Effective management of nutritional challenges in pediatric oncology requires a coordinated, multidisciplinary approach. Depending on the patient′s clinical status, nutritional interventions may include oral dietary supplementation, enteral nutrition, or parenteral nutrition, selected according to individual requirements and treatment tolerance [45]. Ready‐to‐use therapeutic foods (RUTFs) represent a cost‐effective intervention, with randomized controlled trials showing improved weight gain (77.8% vs. 64.2% in controls) and a substantial reduction in severe infections (10.6% vs. 31%) [46]. Implementation entails consideration of local food availability, cultural acceptability, and the capacity of the healthcare system infrastructure [12]. Although protein supplementation interventions have demonstrated potential benefit in several studies, their efficacy must be validated through large‐scale randomized controlled trials. While resistance training with protein supplementation (21 g of whey protein daily) is feasible and safe in childhood cancer survivors, the additional benefits of protein supplementation beyond exercise alone are limited, denoting the need for optimized dosing strategies and timing protocols [47, 48].

Personalized nutritional plans are key to addressing the distinct challenges each patient faces. Nutritional counseling and support play vital roles in management strategies. Collaboration among dietitians, clinical nutritionists, and oncologists is important for developing and implementing effective nutritional care plans [44]. Nurses have a central role in day‐to‐day nutritional monitoring, family education regarding diet changes, and adherence support, functions particularly critical in LMICs where availability to specialized nutrition services may be limited, and nurses frequently serve as primary patient contacts for ongoing care coordination. Additionally, engaging families in the nutritional care process is important, including educating them about the importance of nutrition and involving them in meal planning and preparation [49].

Notwithstanding these interventions and strategies, high‐quality randomized controlled trials may be essential to evaluate the effectiveness of various nutritional interventions across diverse LMIC settings [50]. A consensus statement emphasized the key role of early identification and management of malnutrition in children undergoing cancer treatment [51] and recommended a standardized approach to nutritional support [51]. This exposes a significant research gap and the need for more methodical studies to guide clinical practice.

### 3.6. Research Gaps and Future Directions

Despite the significant body of research showing the prevalence and effect of malnutrition on pediatric cancer in LMICs, several research gaps remain. There is a need for more high‐quality, rigorously designed studies to evaluate the effectiveness of different nutritional interventions [50, 52]. Standardized definitions and assessment methods for NS are needed to ensure consistent data collection and comparison across studies [53, 54]. Additional research is needed to understand the multifaceted connection between malnutrition, socioeconomic variables, and access to healthcare in affecting outcomes for children with cancer [55]. Studies should investigate the long‐term effects of malnutrition on the growth, development, and health outcomes of pediatric cancer survivors [15]. The impact of the gut microbiome on malnutrition among children with cancer needs further investigation, as does the likelihood of specific nutritional interventions to improve gut health and NS [12]. Finally, additional research is needed to develop culturally appropriate and sustainable programs that address malnutrition and improve the availability of comprehensive cancer care in resource‐constrained settings [56]. Dealing with these research gaps may serve as effective strategies to improve the survival and quality of life of children with cancer in LMICs. Integration of palliative care principles supports comprehensive symptom management that directly affects nutritional intake, including pain control, nausea management, and psychosocial support [57]. In LMIC settings where cure may not be achievable for all patients, palliative approaches to nutrition, focusing on quality of life, comfort, and family support instead of solely on weight gain, represent clinically appropriate care goals warranting consideration alongside curative nutritional interventions. This twofold framework acknowledges that nutritional support serves multiple purposes: optimizing treatment tolerance in curative contexts and maintaining respect and comfort in palliative scenarios [57].

## 4. Conclusion

Malnutrition is a significant and endemic issue among children with cancer in LMICs, substantially affecting clinical outcomes and survival rates. The consistently high prevalence of malnutrition across studies highlights the vital need for action [7, 33, 58]. Tackling malnutrition needs a comprehensive approach, including accurate nutritional assessment, efficient interventions, and strategies to improve healthcare access and address socioeconomic disparities. Although promising interventions are available, further study is needed to develop and evaluate effective, culturally appropriate, and sustainable programs for different LMIC settings. International collaboration and resource allocation are critical to overcoming the challenges LMICs face in providing comprehensive and equitable cancer care for children. Integration of palliative care should be recognized as a necessary component of comprehensive care. Only through unified efforts can survival and quality of life for children with cancer in these regions be improved.

## Author Contributions

M.A‐B. and M.L. contributed to the conceptualization and design of this review.

## Funding

No funding was received for this manuscript.

## Disclosure

All authors reviewed and approved the final manuscript.

## Ethics Statement

This manuscript is a narrative review based solely on previously published research findings and does not involve human participants or identifiable data.

## Conflicts of Interest

The authors declare no conflicts of interest.

## Data Availability

Data sharing is not applicable to this article as no datasets were generated or analyzed during the current study.
